# Eradicating female genital mutilation and cutting in Tanzania: an observational study

**DOI:** 10.1186/s12889-015-2439-1

**Published:** 2015-11-19

**Authors:** Moses Galukande, Joseph Kamara, Violet Ndabwire, Elisabeth Leistey, Cecilia Valla, Sam Luboga

**Affiliations:** Department of Surgery, Makerere University College of Health Sciences, Mulago Hill Road, P.O. Box 7072, Kampala, Uganda; World Vision Australia, Melbourne, Australia; World Vision Tanzania, Arusha, Tanzania

**Keywords:** FGM/C, Female genital mutilation and cutting, Intervention, Africa, Tanzania

## Abstract

**Background:**

Female genital mutilation and cutting (FGM/C) has long been practiced in various parts of the world. The practice is still prevalent in 29 countries on the African continent despite decades of campaigning to eradicate it. The approaches for eradication have been multi-pronged, including but not limited to, health risk campaigns teaching about the health consequences for the girls and the women, recruitment of change agents from within the communities and the enforcement of legal mechanisms.

The purpose of this study was to analyse the impact of an 18 month long campaign to eradicate or reduce FGM/C in a rural predominantly Masai community.

**Methods:**

An observational study involving mixed methods, quantitative and qualitative was conducted in Arusha region, Tanzania. A household survey, key informant interviews, focus group discussions, school children's group discussions and project document reviews for both baseline and endline assessments were used. Same tools were used for both baseline and endline assessements. Comparison of baseline and endline findings and conclusions were drawn.

**Results:**

The prevalence of self reported FGM/C at endline was 69.2 %. However, physical obstetric examination of women in labour revealed a prevalence of over 95 % FGM/C among women in labour.

Those in favour of FGM/C eradication were 88 %. Nearly a third of the 100 FGM practitioners had denounced the practice; they also formed a peer group that met regularly comparing baseline and endline. Knowledge about FGM/C health risks increased from 16 to 30 % (*p* < 0.001). The practice is currently done secretly to an uncertain extent.

**Conclusion:**

This multifaceted educational campaign achieved moderate success in increasing knowledge of the health risks and changing attitudes despite a short period of intervention. However, its effectiveness in reducing FGM/C prevalence was uncertain.

## Background

FGM is defined as any procedure that cuts or harms female genitalia without medical indication [1]. In the 29 countries, from which prevalence data does exist, an estimated 101 million girls and women above 9 years have undergone FGM [2], and 3.3 million girls are at risk of being subjected to FGM annually. In these 29 countries the prevalence of FGM ranges from 0.6 % to 98 %, although the practice of FGM is also found in other countries, including among migrants from FGM practicing countries [3].

The prevalence of Female Genital Mutilation (FGM/C) is reduced in almost all countries in which it is a traditional practice [4]. Although there are variations in countries and communities, ranging from no change at all to countries, and communities where the practice has been more than halved within a generation.

Tanzania is one of the 29 countries where FGM is still prevalent [2, 3]. It is estimated that 7.9 million women and girls in Tanzania have undergone FGM [3]. The target area, Arusha, is one of the regions in Tanzania with a high prevalence of FGM.

This paper is an evaluation of a project that aimed at eradicating FGM/C in the Tanzania predominantly Masai community. The project was a public health intervention targeted at behavioural risk factor genital mutilation/cutting. This exercise was preceded by a baseline survey for benchmarking 12 months before the impact evaluation study.

### Setting and study context

This project evaluation study was performed over 18-day duration, in Arusha, Tanzania in a predominantly Masai community with illiteracy rates as high as 50 % for both men and women and high children school dropout rates [5]. FGM/C has been practiced in these communities for hundreds of years and is usually performed on children by traditional practitioners under none sterile conditions [6]. In 1998 a law outlawing FGM/C was enacted in TZ [7]. The Tanzania sexual offenses special provisions Act, a 1998 amendment to the penal code specifically prohibit FGM.

## Methods

### Design

The survey was a cross-sectional study that used mixed methods involving a combination of quantitative methods to establish the attainment of benchmark indicators and qualitative methods to analyze and better understand the contextual situation in the target communities. Mixed methods allowed us to triangulate our findings and to generate a rich and comprehensive picture of the factors affecting FGM in the study area.

In the HH survey, we aimed to sample a minimum of 891 interviews with valid data and a response rate of 90 %. In addition eight Key Informant Interviews were conducted, they included: a Health worker, a member of village health team, the Project coordinator, a community leader, and representatives from local women and men’s groups; 11 FGDs were done, each FGD comprised of 8 to 10 members, the discussion groups comprised of active and ex- FGM practitioners, adult men, adult young women, adult women, adult young women, and village leader; children group discussions in two schools, the school children were stratified by gender (boys and girls) and by class, upper class (Primary 6 and 7) and lower class (Primary 3, 4 and 5) and four site observation visits in two villages were undertaken.

The evaluation exercise was at baseline (before implementation) and endline (after implementation), a study of multi-faceted community activities aimed at eradicating FGM within the eight villages. There was no control group.

### Project intervention

The following activities were conducted over the project period:

In 2013, 19 activities involving five-school club visits, eight community dialogue sessions, 10 trainings and sessions for health risk of FGM involving children, local leaders and FGM practitioners were conducted. Other activities done included Training for trainers’ session and on income generating activities (IGA) training.

In 2014, five training sessions involving the youth, Masai warriors, FGM practitioners were conducted. An exercise to distribute 50 goats and 300 chickens for ex-FGM practitioners was done. Ongoing activities in both years included weekly school club activities, ex-FGM club meetings and quarterly local village advocacy subcommittee meetings. An extraordinary grand meeting involving 1400 participants was held during which 400 unmutilated/uncut girls made a public declaration denouncing FGM and celebrated their status of being uncut.

### Sampling

A sampling list of all villages and sub villages in two wards, Musa and Mwandet in Mukulat district of Arusha region, Tanzania was established for both surveys. There were a total of eight villages made up of 26 sub villages and 2958 households (HHs) in total. The same design and sampling frame had been used for the baseline survey. Each enumerator was assigned 12–14 households to survey per day for four days excluding the pre-test day during which one household was surveyed per enumerator to ascertain the questionnaire’s cultural appropriateness. In total 14 enumerators participated in the HHS. Two villages were enumerated concurrently per day. HHs was visited with the help of local council representatives. Upon entry, introductions were made and verbal consent obtained. The same number of enumerators, community entry methods and sampling methods were used for the baseline survey. A household “mini census” (recording/ counting men and women in the household) was then carried out for men and women separately; the listing was in descending order according to age. They were randomly and respectively chosen using the Kish grid [8]. The HH sampling was systematic, sampling every 5^th^ HH and no replacement was done for non-responses or vacant HH, as that was factored into the sample size calculations by 10 %. The household head or one of the wives/wife in the HH answered the school dropout questions concerning children aged 6–17 years.

### Study variables

The variables which were also the project outcomes included FGM/C prevalence, self reported FGM/C health risks, intention to stop FGM/C and active participation in anti FGM/C activities.

### Survey Instruments and interview guides

For the qualitative components, the interview guides and FGD schedules were developed in collaboration with World Vision Tanzania (WVT) team and adjusted after obtaining feedback from enumerators and their field coordinators on clarity and cultural appropriateness. Children, women, men, FGM practitioners and community leaders were interviewed separately in order to address the project outcomes related to these groups. The baseline survey instruments including the household survey questionnaire which was modified and therefore required that pre-testing is done again.

All the data collection tools were translated into Swahili and back translated into English to ensure consistency of meaning.

### Data collection

The evaluation exercise took 18 days, including eight days of fieldwork, involving 20 trained enumerators and three data entrants. Three enumerator supervisors, one WVT field staff, one lead enumerator with two other assistants monitored quality control. Quality control measures included: The enumerator teams were briefed each morning as they picked their questionnaires and taken through the instructions for emphasis. At the end of each day there was a debrief meeting to discuss misinterpretations and emerging challenges.

The FGD interviewers met with the evaluation team on a daily basis to transcribe FGD notes. All discussions which had been handwritten during the discussions were transcribed verbatim before analysis.

The supervisors and data entrants checked the collected questionnaires, to ensure completeness and accuracy.

#### Eligibility, inclusion and exclusion

In the household surveys we interviewed adults aged 18 and above. Questions about the school children who were aged 6 to 17 years dropouts were answered by an adult in the HH which was preferably the head in the HH. This was based on the assumption that respondents can provide valid information on the experience of their daughters and other young female occupants of the households.

### Data analysis

All quantitative data were summarized using descriptive, univariate and multivariate analyses (where appropriate) using Amos 20.0 (SPSS, Chicago, IL, US) software. Statistical significance was when *p* < 0.05. Qualitative data were inductively analysed using thematic and content analyses with predetermined criteria (a starter list of the initial codes). In the inductive analysis, codes were generated in the course of the analysis, a team of five members compared and discussed coding as well as emerging themes.

Focus Group Discussions: Data were collected in pairs (the pairing was based on one of the interviewers being fluent in a Masai dialect), scripts were re-read and rewrote on the same day of data collection before coding was done using pre-determined criteria. This predetermined criteria were informed by the project key outcomes (see study variables) from which the study variables and questions were derived.

Key Informant Interview: Data were collected by a single enumerator with a translator when and as required; they reread and transcribed the scripts on the same day of data collection before coding was done using pre-determined criteria. This predetermined criteria were informed by the project key outcomes from which the study variables and questions were derived.

### Ethical consideration

All participants provided verbal consent and the study was approved by the Makerere University School of Medicine Research Committee

Verbal informed consent from adults was sought from respondents before interviews. Participants were informed that there were minimal or no risk to their participation in the study, that participation was voluntary, and that they could withdraw their participation anytime during the interview. They were also informed that refusing to participate would not affect the usual services they normally access at the WVT (World Vision Tanzania).

## Results

**Fig. 1:**
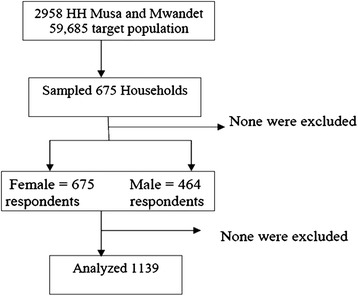
Participants recruitment flow chart

The results presented here are from both the baseline and endline assessments. The different sources of data (household survey, FGDs, KIIs and document reviews are indicated). Figure  1 shows the participants recruited in the study at endline.

**Fig. 2:**
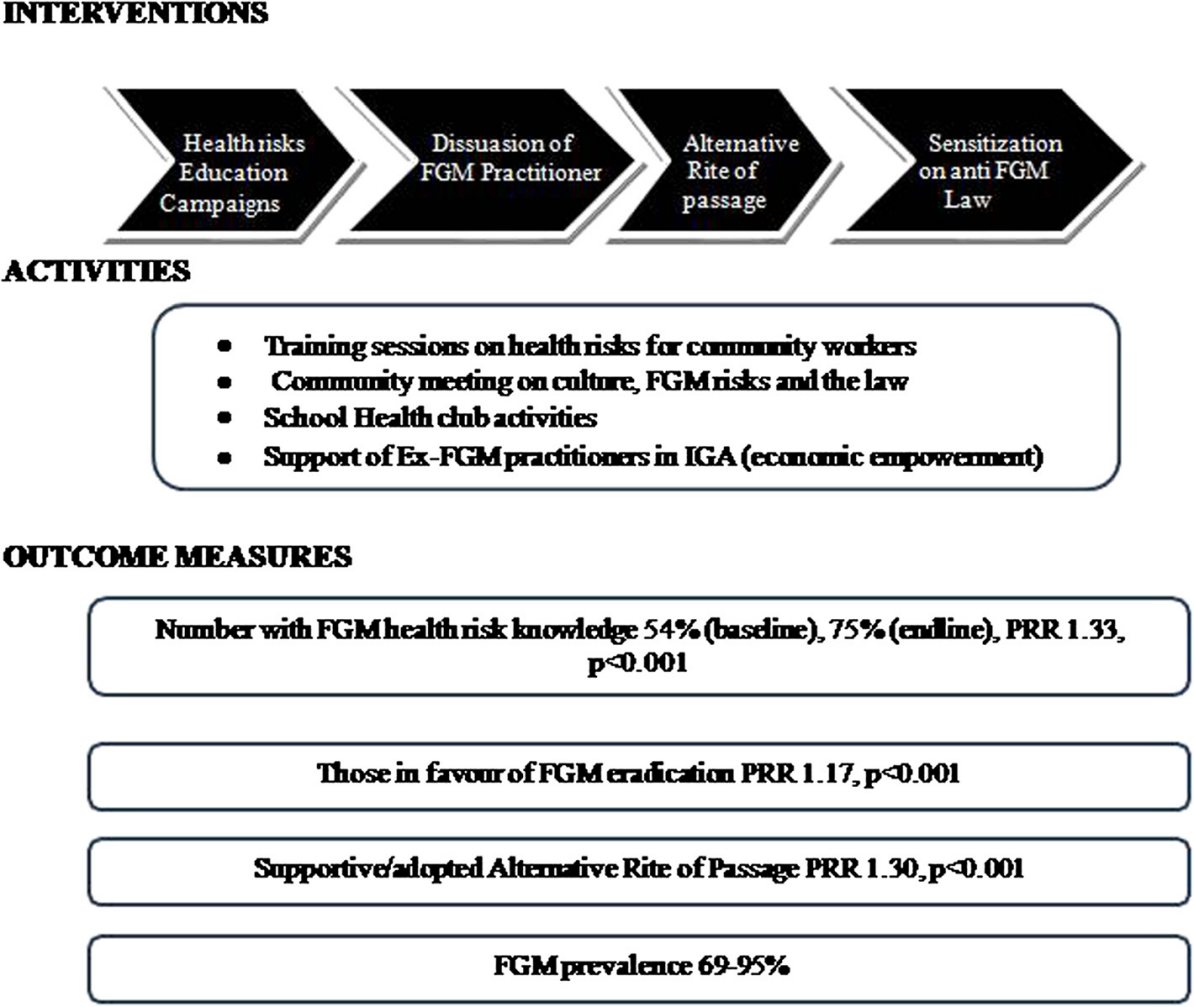
Illustrating project interventions, activities and selected outcomes

Figure 2 summarizes the project interventions which were: FGM health risk educational campaigns, dissuasion of FGM practitioners, offering of a cultural acceptable alternative to FGM (ARP) and increasing awareness of government anti FGM laws. In addition, the activities are shown which mostly included training, meetings, other community and school led activities as well as income generating activities. And finally outcome measures concerning knowledge, attitude change and FGM prevalence are also shown.

In the HHS the mean age of female respondents was 34 years (SD 12), with a range of 18-85y and for men the mean was 43 years (SD 15), with a range of 18-100years. The male to female ratio was1: 2.

42 % of women and 37 % of men had had no schooling, while 51 % and 55 % of women and men, respectively had attained some level of primary school education (see Table 1).

**Table 1:** Mukulat anti female genital mutilation endline household survey participants’ demographics

Variable		Women (*n*, %)	Men (*n*,%)	Subtotal	Total^a^
Age	Mean	34 years	43 years		
	SD	12	15		
Ward					
	Musa	528	345	873	
	Mwandet	147	119	266	1139
Village					
	Engurtoto	176	105	281	
	Imbibia	84	54	138	
	Engalaoni	69	59	128	
	Losikito	199	136	335	
	Olchorovusi	45	23	68	
	Oltushula	21	23	44	
	Likamba	59	40	99	
	Nengungu	22	15	37	1130
Education					
	Never schooled	286	172	458	
	Primary level only	347	253	600	
	Secondary school level only	1	33	34	
	Tertiary	3	6	9	1101
Marital status	Single	27	45	72	
	Married	598	415	1013	
	Divorced/separated	3	1	4	
	Widow	47	0	47	1064

^a^Totals less than 1139 were due to non responses or missing data

All the eight villages and 26 sub villages were surveyed, 1139 individuals were recruited out of a possible 59,685, see Fig. 2.

### FGM prevalence

Out of the 675 female study participants, 467 (69.2 %) respondents reported that FGM/C had been performed on them at varying ages and 107 (15.8 %) respondents had it done under the age of 11 years. There was no similar comparison of data from the baseline. The endline tool was altered to include these data that were missed in the baseline. Ninety five percent of women delivered at the health center had had FGM performed on them evidenced by a pre-delivery (in labour) physical examination and documentation.

### Change in knowledge of FGM related health risks

There was not much difference between the men and women who knew the health risks of FGM/C, 70.4 % and 74.8 % of women and men respectively. There were participants (16.4–17.5 %) that believed FGM/C was beneficial in some ways such as acceptance in society. 26.4 % of women and 22.2 % of men had actively participated in anti FGM activities over the past 18 months. 82.4–83.4 % were in favor of eradicating FGM (see Table 2).

**Table 2:** The key findings from the endline household survey

Variable	Women *n* = 675	Men *n* = 464
Those who stated that girls marry below 18 years in the village	353 (52 %)	248 (53 %)
Women who self-reported undergoing FGM	467 (69.2 %)	-
Age at which FGM had been done		
≤ 11 years	107	-
12–15years	169	-
≥ 16 years	143	-
Those who knew of the FGM health risks	475 (70.4 %)	347 (74.8 %)
Those who believed FGM had positive effects	111 (16.4 %)	81 (17.5 %)
Those that said they had abandoned early marriage practices	623 (92.3 %)	413 (89 %)
Those that knew advocacy subcommittees existed in their area	178 (26.4 %)	103 (22.2 %)
Those with knowledge of children rights and protection laws	277 (41 %)	206 (44.4 %)
Those that said yes to adhering to children rights laws	239 (35.5 %)	199 (42.9 %)
Those optimistic about FGM eradication	547 (81 %)	349 (75.4 %)
Those in favor of eradication	563 (83.4 %)	384 (82.8 %)
Those supportive of ARP without FGM	279 (41.3 %)	168 (36.2 %)
Those with HH members who have participated in anti-FGM	159 (23.6 %)	115 (24.8 %)
Those who answered that the project belongs to the community	538 (79.7 %)	376 (81 %)

*FGM* Female Genital Mutilation/cutting

*ARP* Alternative Rite Passage

*HH* Household

Comparing the HHS baseline and the HHS evaluation, there was a significant increase in the number of those that knew of the FGM/C associated health risks. However, the number of those that participated in anti FGM activities did not increase over the period duration 23.2 % to 24.1 % (*p* < 0.640). The number of community members who thought FGM/C could be dissociated from marriage had increased from 30.2 to 39.2 % (p = 0. 001) see Table 3.

**Table 3:** Comparing some key baseline and endline household survey findings

Project Outcomes	KAP Variables	HHS Baseline *n* = 1013	HHS Endline *n* = 1139	*P* Value	PRR^a^	CI
Community empowerment	Those who knew FGM effects	54 % (536)	72 %(822)	<0.001	1.333	1.50–2.30
	Those in favour of FGM eradicated	62 % (627)	74.4 %(847)	<0.001	1.170	1.10–1.240
	Those who had participated in anti FGM campaign activities	23.2 % (235)	24.1 % (274)	0.6402	1.037	0.80–1.210
ARP Approach	Those who thought culture could still be maintained without FGM	16.6 % (168)	30 % (302)	<0.001	1.600	0.65–1.34
	Those who thought girls can be prepared for marriage without undergoing FGM	30.2 % (306)	39.2 %(447)	<0.001	1.298	1.154–1.46
Alternate IGA for FGM practitioners	Those who knew FGM practitioners that left practice and engaged in other IGA	28.4 % (288)	-	-	-	

^a^PRR – Prevalence rate Ratio showing the number of times follow-up rates are higher/lower relative to the baseline

IGA – Income generating activities

From the KII (by informants who were present and participated in the baseline and the project), there was a general impression that knowledge of FGM effects was increased so was knowledge of children’s rights.

From the FGD, overall the majority, 61/84 (73 %) knew about the negative effects of FGM, such as bleeding, out of the 23 that did not know or said they did not know the negative side effects of FGM were from a single group of 12 active FGM practitioners from Engalaoni in Baombani. A further 8 young women aged 18–24 year too did not know the negative effects of FGM. Those that knew of the negative effects cited trainings as the source of this knowledge and change of mind.

### Dissuasion of FGM practitioners and community abandonment of FGM

Some of the key informants stated that a number of FGM practitioners had declared publicly that they had given up the FGM practice.

Some indicated that ex-FGM/C practitioners were still carrying out FGM practices. Most boys suggested they would marry a girl who wasn’t mutilated. A significant proportion of the children were unaware of the law against FGM (see Table 4).

**Table 4:** Responses for 154 individual school children from two primary schools in Mukulat (Engalaoni and Nengungu) endline survey

Gender	Class	Number	Knew FGM Health risks	Yes to FGM eradication	Marry uncut	Knew of girls undergoing ARP	Awareness of Anti-FGM Tanzania law	Knew of girls who rejected FGM^b^	Knew of FGM practitioners who publicly rejected FGM^c^
Girls	P6	12	12	12	12	A few	Nil	None	None
	P5	24	24	24	24	02	Nil	None	None
	P4	24	24	24	24	12	Nil	None	None
	P3	24	24	24	24	05	Nil	None	2 knew of them
		80	All knew	All said yes	All said yes	All were not aware	None were aware	-	-
Boys	P6	24	24	24	24	Not heard of it	0	24	4
	P5	0	-	-	-	-	-	-	-
	P4	22	24	20	15^a^	0	22	22	6
	P3	24	24	24	24	0	1	24	0
		70							

^a^2 said they won’t marry uncut woman and 5 did not declare any opinion

^b^All the boys stated that even though the girls objected to FGM/C, they are forced or coerced, punished and shamed

^c^A few stated that they knew of ‘Ex-FGM practitioners that are back to practicing

P stands for primary class

From the FGDs to the question, ‘Would you allow your son to marry an un-cut woman?’ Overall, 61 (64 %) said yes, they would allow, and 35 said no. Of those saying yes, 40/61 were from younger age bracket (18–35 years).

70 % of those who said yes were from peri-urban villages. Some of the reasons given were that boys are free to decide, besides, they may run away from the home if things were forced on them.

To the question, ‘do you support eradication or discontinuation of FGM?’ 45/70 (64 %) said yes (24 women and 21 men, there were no significant differences between men, and women, young and old, though this is less than in the HH.

Those saying yes, attributed it to the trainings they had with WVT, learning about the negative effects of FGM. One participant, however, cautioned that not all who say yes mean yes.

To the question, ‘is it possible to eradicate FGM?’ 52 (81 %) out 64 said yes. The 12 who said no were from a group of 12 practitioners from Engalaoni, the reason for lack of optimism was uncertain.

From the HHS there was high optimism about the eradication of FGM and those in favor of it were over 80 % (see Table 3).

From the children discussion groups also notable was that all the girls were not willing to be cut, but were coerced into the practice using several methods including violence.

A number of FGM practitioners approximately 33 denounced FGM during the project period, although an equal number or more exist in the Musa and Mwandet area. The exact number of FGM practitioners is not known. Whereas a happy number denounced FGM, we were not certain how many sustained the intention and not ‘backslid’ into the practice. The data from the children's discussions was suggesting attrition.

A large meeting of more than 400 girls met and celebrated being uncut; it was a broadcast on International Televisions in Africa and Europe in early 2014. The WVT program officer stated “… this is the most successful thing I have ever done”.

**Fig. 3:**
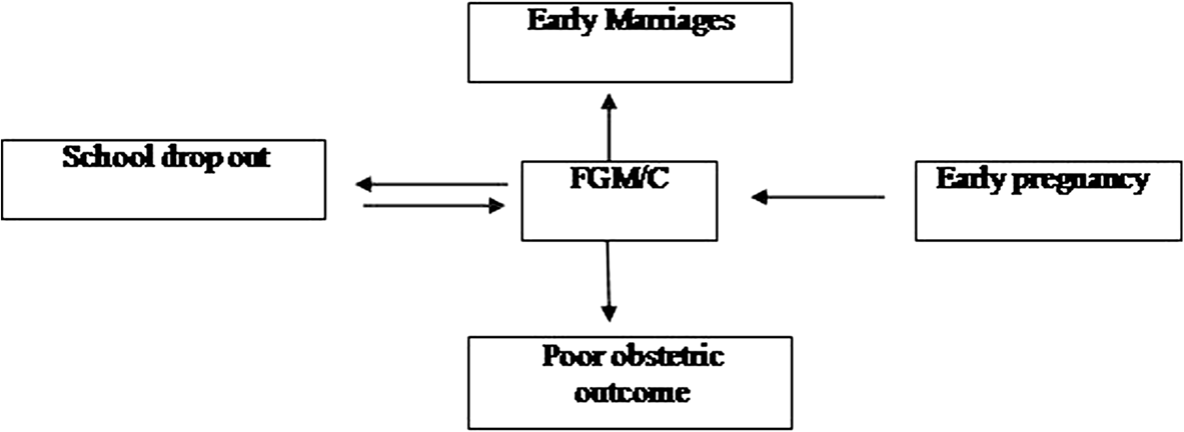
Illustrating the linkages, factors and drivers of school children drop outs

Early marriages are still a problem, aggravated by a high school dropout rate in Musa and Mwandet and also triggered by FGM and teen pregnancy see Fig. 3.

Most key informants stated that FGM is likely to still be active and underground. Women are cut during or after delivery and also ‘small’ girls who are unable to report or talk about it are increasingly targeted. On a separate occasion it was reported that a 16 year old girl who was refused by her parents to be cut, self-mutilated so that she would marry a boy who had threatened not to marry her because she was uncut. Figure 3 relates some of the interplay between early marriage, pregnancy and FGM/C.

Some key informants reported that some FGM practitioners may be taking the chicken and goats, but still carry on practicing FGM, simply because they get much more money from these activities more than from what WVT offers.

From the FGDs there were suggestions what is given may not be sufficient to dissuade practitioners for a long time’. It was reported that FGM is now done secretly, because it is criminalized. …”FGM is still going on but not celebrated,” reported by 8 adult men over 40 years in Likamba.

Seven young women in Shuleni said that…” it's done secretly now days” because of these anti FGM activities we now do it during childbirth without prior warning or consent.

Some or many FGM practitioners double as traditional Birth Attendants (TBAs).

School girls are cut during school holidays so that they have enough time to heal and recover. So long school holidays should be looked out for.

From the children discussion some mentioned FGM practitioners that denounced, but still acted secretly, this collaborates with the views from some of the KII and FGDs.

### Sensitization on anti-FGM Law

From the HHS those with self-reported knowledge of the laws and policies protecting children's rights were 41 % for women and 44 % for the men (see Table 3). There was no direct comparator from the baseline survey.

From the school children's group discussions, the number of children that showed knowledge of FGM effects was high see Table 4. The commonly cited effects included; excessive bleeding, difficult childbirth, painful intercourse, death and catching diseases like HIV. Though most of these pupils are unaware of the law, some said they knew WVT and the government are against FGM but are not aware of any law

All key informants expressed the views that eradication was achievable, but required a sustained campaign against FGM. This education campaign teaching about the negative effects of FGM and sensitizing about what the anti FGM law.

### Alternative rite of passage (ARP) as an option to FGM

From the FGDs to the question, ‘is ARP sufficient without FGM?’ 49/83 (59 %) responded yes. Both the young men and women said yes. The reason given was for those who said no was that ‘the women cannot be taught to be women without being cut’. Some said they wished not to be cut. Those who said no said ARP is not happening.

One stated that, WVT is here only for a time what will happen when they go yet this practice guarantees a continuous livelihood.

To the question, ‘should early marriage be abandoned?’ Most respondents yes 70/82 (85 %), the reasons they gave were that young girls are not old enough to take on the responsibility of looking after a home.

The FGM practitioners said they prefer to circumcise older girls above 20 years, because it is easier and may not lead to serious adverse events. They, however, stated that it is not up to them, it is the families that summon them to cut. Also early marriage denies girls the opportunity to go to school.

An observation by some of the respondents was made that there are more marriages now than before.

From the HHS there was a significant difference between the numbers of respondents who thought that de-linking FGM from culture and marriage readiness for young girls was possible.

Whereas 36-41 % were supportive of ARP the data from KII and FGD indicated that this is not happening. Some of the people said they didn’t want to pretend that they were doing FGM while they were not.

In the school children's group discussions, all girls were not in favor of FGM/C and were in acceptance of alternative rite passage (ARP).

## Discussion

We set out to evaluate the impact of an intervention campaign against FGM/C. We found significant positive changes in knowledge about the health risks of FGM/C. There was also significant increase (compared to baseline) in the number of community members that were in favor (positive intentions) of eradicating FGM/C. What wasn’t certain was the extent of decrease in FGM/C prevalence.

Tanzania is one of the 29 countries where FGM is still prevalent [2, 3]. It is estimated that 7.9 million women and girls in Tanzania have undergone FGM. The target area, Arusha, is one of the regions in Tanzania with a high prevalence of FGM. In this survey, prevalence was 69-96 %, way above the national average of 14.6 %. Combining the 2005 and 2010 demographic, household survey (DHS) data, the prevalence from in this area rose, 54.5 % to 58.6 %.

The FGM/C practice is related to ethnicity [9] and in the area where this intervention was carried out is predominantly Masai. The other associated risk factors are poverty, low education and rural residence [6, 10]. In this study this area was rural with illiteracy rates as high as 50 % for both men and women. In the last two DHS in Tanzania, conducted in 2005 and 2010 respectively, the prevalence of FGM/C is higher among the less educated [11, 12] with 3.1 % among those with secondary level of education and 20.3 % among those with no education.

### The intervention

The intervention was multifaceted, concentrating on the health risk education approach, social economic development, the use of change agents from within the community (FGM Practitioners) encouraging alternative rite of passage ARP, legal mechanisms and formal school education. These approaches have been described before [3, 14]. The target population was close to 60,000 people, over the 18 month period. 25 % of this population was reached (they participated in anti FGM activities). Only one WVT field officer supervised the project, but drew considerable support from the community human resource to implement the project plan. This was a deliberate strategy to ensure that the community owns the project and therefore enhance long term sustainability. Indeed, in the HHS over 90 % indicated that they viewed this as owned by them (community).

### Impact

This campaign appeared to produce positive effects or improvements in knowledge or beliefs about FGM/C as evidence by: 30 FGM practitioners had denounced the practice out of an estimated 100; they formed a peer group that meets regularly.

88 % of the study participants in the Household survey were in favor of discontinuation of FGM/C, their traditionally entrenched practice. The percentage of those knowing about the negative health risks of FGM/C had increased from 54 % (baseline survey) to 75 % (endline) in one year (*p* < 0.001).

There were signs of attitudinal change too: The percentage of those who think that FGM can be disassociated from the culture had increased from 16 % (baseline survey) to 30 % (*p* < 0.001) and 64 % of men and women in FGD said they would allow their son to marry uncircumcised women.

It was not clear whether community empowerment positively affected (behavior) the prevalence of FGM/C, as there was no comparison (reference) rate in the baseline HHS survey. However, the self reported FGM prevalence among adult women interviewed in the HHS was 69.2 %. Of those cut, 16 % were under 11 years. 25 % were aged 12–15 years and 21 % were 16 years or older.

In the 2010 district health survey, the cutting of young girls under or equal to 1 year had increased from 18.4 to 31.7 % compared to the 2005 district health survey. The explanation for this trend change is attributed to the abrupt nature of attempts to abolish FGM/C that was started in the 1970s.

The key informant at a health facility where the community women deliver babies indicated that over 90 % of them had been cut. These data were collaborated with what was in the operating room registers. They showed that nearly all the women (96 %) who had delivered there in the months of August and September 2014 had been cut. It is worth noting that the self reported rate of FGM/C (69.2 %) was less than the 96 % revealed during the obstetric physical examinations. This suggests that the respondents under reported. This could be explained by the courtesy bias or fear of the law.

There is a real danger that FGM activities have gone underground, this view is shared by other researchers [4, 13–15]. The beginning of this phenomenon could have preceded this project inception and perhaps sparked off by the enactment of the anti FGM law in Tanzania. A significant number of respondents indicated that cutting is being subjected more to girls younger than 10 years and in some instances neonates. This was supported by the some KII and FGD findings.

The school dropout rates in Musa and Mwandet wards for both girls and boys were up to 40 %. In Engalaoni a rural village predominantly inhabited by the Masai the dropout rate for girls in Standard (Primary) 7 was 66 % between January to July 2014 [ Arusha District Department , school enrollment and drop out data report, 2014, unpublished ]. 

School drop outs, early pregnancy, early marriage can all be triggered by FGM/C and all these triggering each other as was illustrated in Fig. 3 and impact on obstetric outcome [16] .

The public health intervention to eradicate FGM/C seemed to have realized modest gain. These gains were realized at individual and community levels. At individual level through enhancing individual knowledge and motivation and at community level through sustained advocacy campaigns. This approach is based on the premise that interventions to change health related behavior should not only be limited to individual capability and motivation, but also to social, cultural and economic factors [17].

### Limitations

This study was not without limitations: The baseline HHS was conducted 6 months after the commencement of the project. Therefore the changes measured during the evaluation exercise exclude this 6 month period. This may therefore have led to an underestimation of the intervention impact.

It may not be entirely possible to attribute the changes observed in the survey to the intervention because there was no control group. In this situation, it is plausible that we may have overstated the impact of the intervention. Nevertheless, some important indicators had more than doubled and these were attributed to the effort put into sensitizing, educating and supporting child rights protection.

Although the data collection tools had a Swahili translation, some respondents were not fluent in Swahili and required a translator in a Masai dialect. This could have potentially misrepresented some of the questions in the tool(s).

The HHS prevalence of FGM/C was self-reported; it is plausible that there would have been under reporting perhaps due to experiment effect or social desirability.

We assumed that the household heads or adults found in the household would know and give valid responses to the school dropout questions concerning children aged 6–17 years. We anticipated the household heads would know the FGM/C experiences of their household members.

### Moving forward

We suggest an emphasis be put on the further engagement of the traditional leaders’ council, which has the power to make culturally binding decisions.

Other NGOs with gender and children protection and rights focus may be encouraged to include anti-FGM activities alongside what they do in the same communities targeted by WVT.

Although multi faceted, this was a largely health, educational campaign aimed at imparting knowledge, changing attitudes and ultimately behavior. Therefore, emphasis on future interviews should be on improving the competence of the persons who deliver these educational sessions. Education may improve knowledge, therefore enhancing individual capabilities and motivation for behavioral change. Competences of adult education (including but not limited to, content preparation, use of visual aids, and appraisal of learning) should be focused on. This may be done in the form of a training of trainers’ workshop (ToT), a needs assessment for these trainers would precede such a ToT workshop(s). Other approaches that encourage behavior change should continue, such as involving all possible change agents and other society champions or celebrities to encourage the community discussing them from FGM harmful practices.

The communities should be empowered to perform periodic mini evaluations of project progress using simple self-assessment tools. They may need prior training to do that, but this may improve ownership and sustainable change.

## Conclusion

This study provides evidence to indicate that a multi-faceted community based approach increased knowledge of health risks associated with FGM/C and changed attitudes, towards this practice. It also suggests that community owned education interventions are feasible in resource poor communities like Mukulat and shows promise in addressing an entrenched violent cultural practice. We encourage sustained effort to effect FGM/C eradication in traditional societies like the Masai of Arusha.

## Abbreviations

ARP: alternative rite passage
DHS: Demographic Health Survey
FGD: Focus Group Discussion
FGM/C: female genital mutilation/cutting
HH: household
HHS: household survey
HHs: households
IGA: income generating activities
KAP: knowledge attitudes and practice
KII: Key Informant Interview
WTA: World Vision Australia
WVT: World Vision Tanzania
WVT: World Vision Tanzania

## References

[1] World Health Organisation. Eliminating Female genital mutilation. An interagency statement, OHCRH, UNAIDS, UNDP, UNECA, UNESCO, UNFPA, UNHCR, UNICEF, UNIFEM, WHO: 2008. http://www.un.org/womenwatch/daw/csw/csw52/statements_missions/Interagency_Statement_on_Eliminating_FGM.pdf.

[2] Female genital mutilation/cutting: estimates of numbers from national surveys in 29 countries with national surveys. http://www.unicef.org/media/files/FGM-C Report 7 15 Final LR.pdf.

[3] UNICEF. Progress for children, A Report Card on Child Protection 8, 2009. www.unicef.org/progressforchildren/ Updated: 18 December 2013

[4] Yoder S, Wang S, Johansen REB. Female genital mutilation/cutting: estimates of numbers from national surveys in 28 countries with national surveys, Studies in Family Planning. In press.

[5] Children and Women in Tanzania, 2010. http://www.unicef.org/tanzania/SITAN_Mainland_report.pdf Accessed April 2015.

[6] Morison L, Scherf C, Ekpo G, Paine K, West B, Coleman R, Walraven G (2001). The long term reproductive health consequences of female genital cutting in rural Gambia: a community based survey. Trop Med Int Health.

[7] Female Genital Mutilation Practice in Tanzania. http://wgc.womenglobalconnection.org/cmf606proceedings.

[8] Lewis-Beck MS, Bryman A, Futing Liao, T. The SAGE Encyclopedia of Social Science Research Methods 2004. ISBN: 9780761923633 doi:http://dx.doi.org/10.4135/9781412950589

[9] Ending female genital mutilation in the UK. http://www.thelancet.com/pdfs/journals/lancet/PIIS0140-6736(13)62353-3.pdf .

[10] Almroth L, Elmusharaf S, El Hadi N, Obeid A, El Mohamed SAA, Elfadil MS (2005). Primary infertility after genital mutilation in girlhood in Sudan: a case–control study. Lancet.

[11] National Bureau of Statistics, Dares Salaam, Tanzania. Tanzania Demographic Health Survey 2004–2005, National Bureau of Statistics, Dares Salaam, Tanzania and ICF Macro, Calverton, Maryland, USA. http://www.measuredhs.com/publications/publication-fr243-dhs-finalreports.cfm.

[12] National Bureau of Statistics, Tanzania. Tanzania Demographic and Health Survey 2004–2005, National Bureau of Statistics, Ministry of Planning, Empowerment and Economics www.nbs.go.tz/tnada/index.php/catalog/8. Accessed October 2014.

[13] Denison E, Berg RC, Lewin S, Fretheim A. Effectiveness of interventions designed to reduce the prevalence of female genital mutilation/cutting. Report from Kunnskapssenteret (Norwegian Knowledge Centre for the Health Services) No 25–2009: http://apps.who.int/rhl/reviews/FGMSR0925genitalmutilation.pdf. 29320117

[14] FGM Country profile Tanzania. www.28toomany.org. Accessed October 2014.

[15] Johansen REB, Diop NJ, Laverack G, Leye E. What works and what does not: A discussion of popular approaches for the abandonment of FGM. Obstet Gynecol Int. 2013. doi:10.115s/2013/348248. 10.1155/2013/348248 PMC3655658 23737795

[16] WHO study group on female genital mutilation and obstetric outcome (2006). Female genital mutilation and obstetric outcome: WHO collaborative prospective study in six African countries. Lancet.

[17] Glanz K, Bishop DB (2010). The role of behavioral science theory in development and implementation of public health interventions. Annu Rev Publ Health.

